# The Evaluation of the Effectiveness of Biomineralization Technology in Improving the Strength of Damaged Fiber-Reinforced LWAC

**DOI:** 10.3390/ma17010214

**Published:** 2023-12-30

**Authors:** How-Ji Chen, Tsung-Kai Chen, Chao-Wei Tang, Han-Wen Chang

**Affiliations:** 1 Department of Civil Engineering, National Chung-Hsing University, 145 Xingda Rd., South District, Taichung City 40227, Taiwan; hojichen@dragon.nchu.edu.tw (H.-J.C.); ru9378@gmail.com (T.-K.C.); awiuyg58799@yahoo.com.tw (H.-W.C.); 2 Department of Civil Engineering and Geomatics, Cheng Shiu University, No. 840, Chengching Rd., Niaosong District, Kaohsiung 83347, Taiwan; 3 Center for Environmental Toxin and Emerging-Contaminant Research, Cheng Shiu University, No. 840, Chengching Rd., Niaosong District, Kaohsiung 83347, Taiwan; 4 Super Micro Mass Research and Technology Center, Cheng Shiu University, No. 840, Chengching Rd., Niaosong District, Kaohsiung 83347, Taiwan

**Keywords:** biomineralization, lightweight aggregate concrete, compressive strength, bond strength

## Abstract

Concrete cracks and local damage can affect the bond performance between concrete and steel bars, thereby reducing the durability of reinforced concrete structures. Compared with general concrete crack repair methods, biomineralization repair not only has effective bonding capabilities but is also particularly environmentally friendly. Therefore, this study aimed to apply biomineralization technology to repair damaged fiber-reinforced lightweight aggregate concrete (LWAC). Two groups of LWAC specimens were prepared. The experimental group used lightweight aggregates (LWAs) containing bacterial spores and nutrient sources, while the control group used LWAs without bacterial spores and nutrient sources. These specimens were first subjected to compression tests and pull-out tests, respectively, and thus were damaged. After the damaged specimen healed itself in different ways for 28 days, secondary compression and pull-out tests were conducted. The self-healing method of the control group involved placing the specimens in an incubator. The experimental group was divided into experimental group I and experimental group II according to the self-healing method. The self-healing method of experimental group I was the same as that of the control group. The self-healing method of experimental group II involved soaking the specimen in a mixed solution of urea and calcium acetate for two days, and then taking it out and placing it in an incubator for two days, with a cycle of four days. The test results show that in terms of the relative bond strength ratio, the experimental group II increased by 17.9% compared with the control group. Moreover, the precipitate formed at the cracks in the sample was confirmed to be calcium carbonate with the EDS and XRD analysis results, which improved the compressive strength and bond strength after self-healing. This indicates that the biomineralization self-healing method used in experimental group II is more effective.

## 1. Introduction

**Figure 1:**
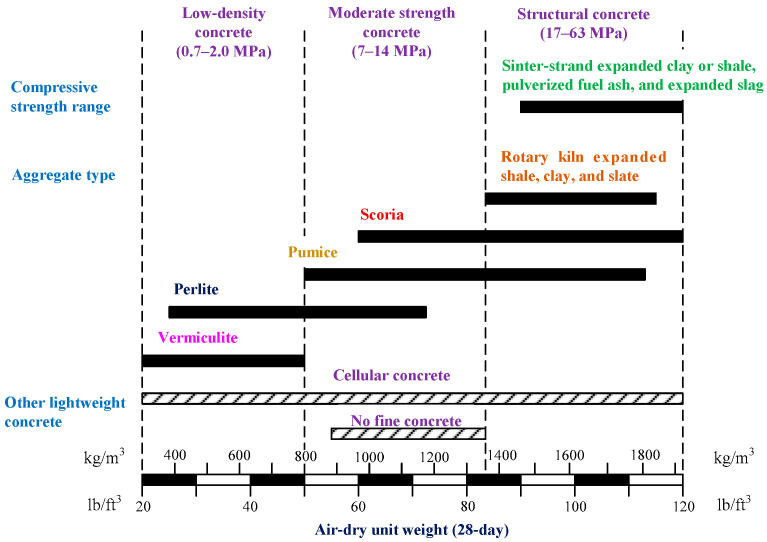
Unit weight and classification of lightweight concrete.

Lightweight aggregate concrete (LWAC) generally refers to concrete produced by replacing normal-weight aggregates with lightweight aggregates (LWAs) with a smaller unit weight or specific gravity [1]. ACI divides lightweight concrete into three grades: low-density concrete, medium-strength concrete, and structural concrete, as shown in Figure 1 [2]. The use of structural LWAC can not only reduce the weight of the structure, but also effectively reduce the seismic load of the building structure [3,4,5]. However, compared with normal-weight concrete (NWC) of the same compressive strength, LWAC exhibits higher brittleness and lower mechanical properties [6,7]. In view of this, scholars believe that using fibers in LWAC is a suitable solution for such problems [8,9,10]. In the past two decades, with the continuous improvement in artificial LWA production technology and the use of fibers, the mechanical properties of fiber-reinforced LWAC has been significantly improved. As a result, the application of LWAC in structural concrete is more common [11,12].

The bond slip characteristics between steel bars and LWAC are the main mechanism to enhance the load-bearing capacity and coordinated deformation of LWAC components and can be used to analyze the mechanical properties of its key parts [13,14,15]. The ACI 408-03 specification [16] states that efficient and reliable load transfer from steel reinforcement to surrounding concrete is necessary for the optimal design of reinforced concrete (RC) structures. According to the definition of ACI 318 [17], bond stress is the shear stress transmitted along the steel–concrete interface. Due to the lower strength of LWA, many studies have pointed out that, at the same compressive strength level, the bond strength between LWAC and steel bars is worse than that between NWC and steel bars [18,19,20]. For this reason, ACI 318-19 recommends a development length correction factor of 1.3 for steel bars to reflect the lower mechanical strength of LWAC compared to NWC of the same compressive strength [17]. However, many studies show different results [4,21,22,23]. Mo et al. [21] showed that the relatively high cementitious material content and the excellent interlocking effect of LWA jointly improve the bond strength of LWAC.

**Figure 2:**
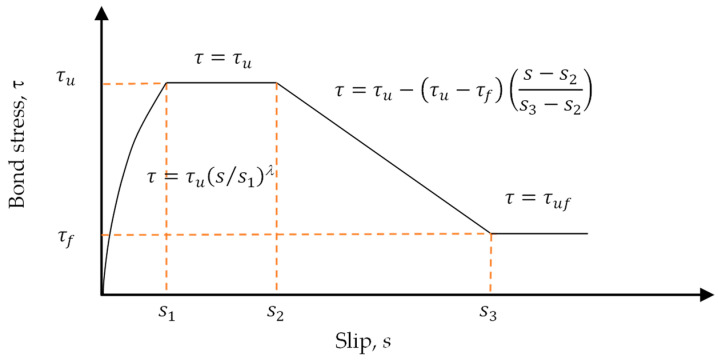
Analytical bond stress–slip relationship.

Many studies have investigated the bond stress between steel bars and concrete, from which it can be concluded that it is related to several parameters [24,25,26,27,28]. For example, the compressive strength of the concrete, the roughness of the steel bar surface, the diameter of the steel bars, and the type and configuration of the ribs. The CEB-FIP Model Code 1990 [29] is based on these research results. It includes four different branches: the curve rising stage, the constant maximum stage, the linear decreasing stage, and the constant friction adhesive stress stage, as shown in Figure 2. In the case of pull-out failure, the bond stress (*τ*) between concrete and steel bars as a function of relative displacement (*s*) can be calculated using the following equation [29]:

(1) $$\tau =\tau_{u}{{\left(s/s_{1}\right)}^{\lambda }}^{}\;for\;0\leq s\leq s_{1}$$

(2) $$\tau =\tau_{u}\;for\;s_{1}<s\leq s_{2}$$

(3) $$\tau =\tau_{u}-\left(\tau_{u}-\tau_{f}\right)\left(\frac{s-s_{2}}{s_{3}-s_{2}}\right)\;for\;s_{2}<s\leq s_{3}$$

(4) $$\tau =\tau_{f}\;for\;s_{3}<s$$

where $\tau_{u}$ and $\tau_{f}$ are the peak bond stress and the residual bond stress, respectively; $s_{1}$, $s_{2},$ and $s_{3}$ are the slip at the start of peak bond stress, slip at the end of peak bond stress, and slip at the start of residual bond stress, respectively; and *λ* is a curve-fitting parameter.

In view of the complex mechanisms of bond stress and slip, the bond-slip relationship is generally expressed by piecewise equations based on experimental results [25,26,29,30,31]. However, due to the dispersion of concrete materials and different testing conditions, the key points of the bond–slip relationship proposed by different researchers are quite different [32,33,34]. For example, the bond strength is mainly described by $\alpha {\left(f_{c}^{\prime }\right)}^{\beta }$, while the variation ranges of α and β are 2.5 to 3.5 and 0.5 to 1, respectively. Furthermore, the peak bond stress and residual bond stress of LWAC are different from those of NWC, as shown in Table 1.

Under the action of load and environment, concrete will deform [10]. These deformations often lead to cracking or localized damage to the concrete, adversely affecting its impermeability, resistance to chloride ion attack, and resistance to carbonation. Cracks or local damage to RC components can easily lead to the loss of the bond force between steel bars and concrete [35]. Most of the materials currently used to repair concrete cracks are epoxy resin systems, acrylic resins, or organosilicon polymers [36,37]. However, from an ecological conservation point of view, these materials are less friendly to the environment. In view of this, ecologically sound and sustainable biological system restoration technologies have become viable alternatives [38,39,40,41,42,43,44,45,46,47,48,49]. Biomineralization is a widely occurring effect in nature [38]. Concrete self-healing technology based on biomineralization mainly refers to the use of microbial metabolism to generate insoluble compounds to achieve the self-healing effect of concrete materials. Microbiologically induced calcium carbonate precipitation (MICP) is ubiquitous in nature; that is, specific microorganisms can react with their own life activities to mineralize and form calcium carbonate crystals (CaCO_3_), and deposit them on the surface of bacterial cells [39]. These calcium carbonate crystals exhibit high strength and stability, filling the gaps between particles and binding them together as a cementing material. When bacteria are added directly to concrete, the highly alkaline environment can easily lead to the death of such unprotected bacteria [40]. Therefore, appropriate bacterial carriers must be selected [41]. At present, carriers are mainly divided into natural carrier materials and synthetic carrier materials [42]. Jonkes et al. [43] studied the ability of alkali-resistant spore-forming bacteria to repair concrete cracks and confirmed that the potential application of bacterial spores as self-healing agents seems promising. MICP is a bio-geochemical process with excellent environmental protection and durability properties and shows excellent compatibility with cement-based materials. It can strengthen or repair cement-based materials to improve their pore structure and repair concrete cracks. Compared with physical strengthening and chemical strengthening methods, MICP technology has potential advantages for repairing concrete cracks.

Cracks or local damage to concrete often lead to a decrease in its mechanical properties, which, in turn, affects its bond behavior with steel bars, ultimately resulting in a decrease in the durability of reinforced concrete structures. Most of the existing literature focuses on the self-healing of concrete cracks by MICP. There is no study on the recovery of damaged bond strength between steel bars and the self-healing LWAC matrix. Given this, the purpose of this study is to apply biomineralization technology to improve the strength of damaged fiber-reinforced LWAC. In this study, fiber-reinforced LWAC specimens were prepared in the control group and the experimental group. The fiber-reinforced LWAC in the experimental group used LWAs as bacterial carriers to increase the survival probability of bacteria. The fiber-reinforced LWAC in the control group did not contain bacterial spores. For each group of concrete specimens, planned tests included compressive strength and pull-out tests. Furthermore, the precipitates formed at the cracks of the biomineralized repaired LWAC samples were analyzed using a crack width meter, a field emission scanning electron microscope, an X-ray energy spectrometer, and an X-ray diffractometer.

## 2. Experimental Procedure

### 2.1. Materials

**Figure 3:**
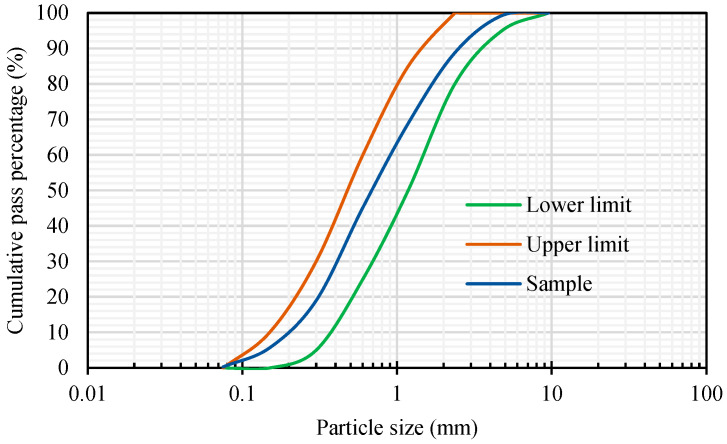
Particle size distribution curve of fine aggregate.

**Figure 4:**
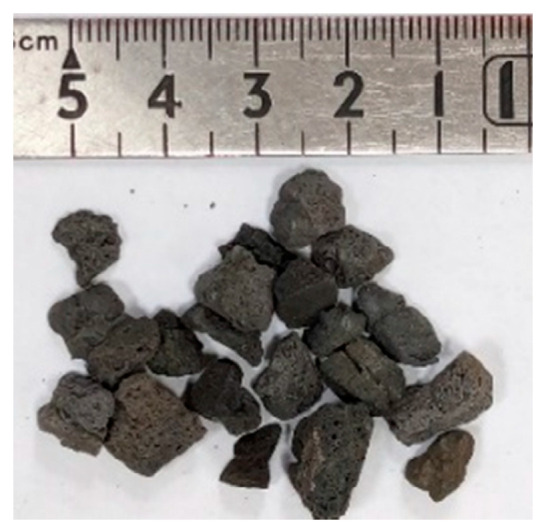
Appearance of expanded shale LWAs.

**Figure 5:**
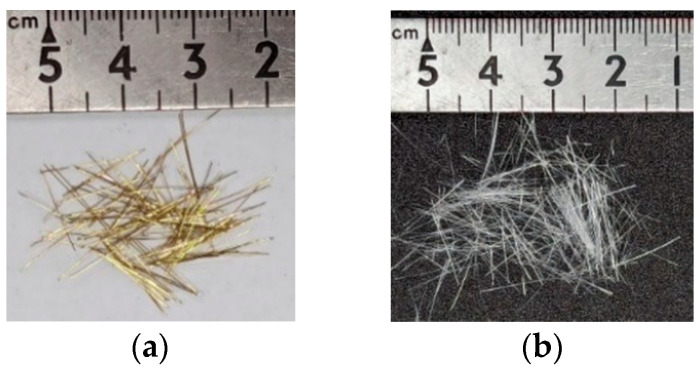
Fibers used in this study: (**a**) short micro-steel fibers and (**b**) polypropylene fibers.

The materials used in this research were as follows:

- *Sporosarcina pasteurii* is a Gram-positive aerobic bacterium. It was provided by Moji Technology Co., Ltd., Chiayi City, Taiwan.
- Calcium lactate was a nutritional source for *pasteurii*. It was purchased from Huacheng Industrial Raw Materials Co., Ltd., Taichung City, Taiwan.
- Yeast extract (YE) is the concentrated content of yeast cells. It can be used as a nutritional supplement. It contains a large amount of protein, amino acids, and the vitamin B group. It was purchased from Huacheng Industrial Raw Materials Co., Ltd.
- During the self-healing process of the specimens, calcium acetate acted as a supplementary source of external calcium ions. It was purchased from Huacheng Industrial Raw Materials Co., Ltd.
- Urea was purchased from Huacheng Industrial Raw Materials Co., Ltd.
- The cement was Type I Portland cement locally produced, with a specific gravity of 3.15 and a fineness of 3550 cm^2^/g; its chemical composition is shown in Table 2.
- The water was local general tap water.
- The fine aggregate was a natural river sand with an FM value of 2.7 and a 24-h water absorption rate of 1.15%. Based on the average of three samples, its particle size distribution curve is shown in Figure 3.
- The LWA was an artificial aggregate whose raw material was shale, as shown in Figure 4. Its basic properties are listed in Table 3. The original maximum particle size of the LWA was 19 mm; the crushed maximum particle size of the LWA was 9.5 mm.
- The superplasticizer was a local product; its chemical composition was water-modified polycarboxylate, and it met the requirements of C494/C494M-17 [50] Type F.
- The fiber was a local product. Short micro-steel fibers (according to ASTM A820/A820M-06 [51]) and polypropylene fibers were used, as shown in Figure 5. The basic properties of the two fibers are shown in Table 4.
- For the longitudinal main reinforcement of the pull-out, #6 rebar was used. Its physical and mechanical properties are shown in Table 5.

### 2.2. Strain Implantation

**Figure 6:**
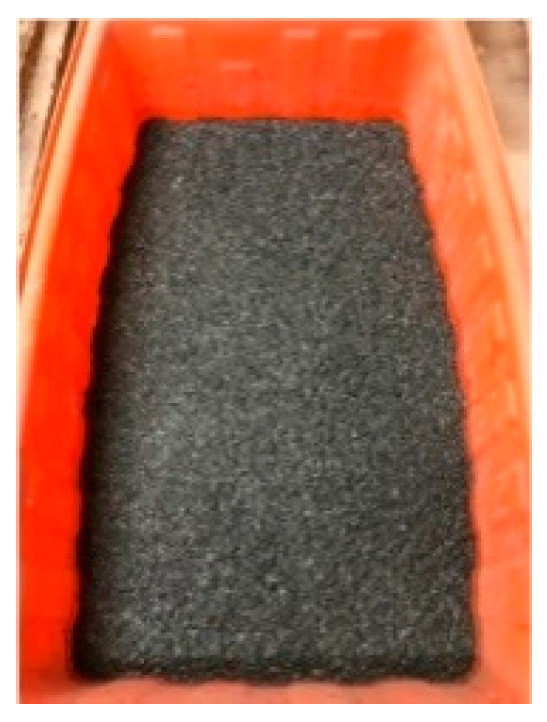
Image of LWAs immersed in a nutrient solution.

**Figure 7:**
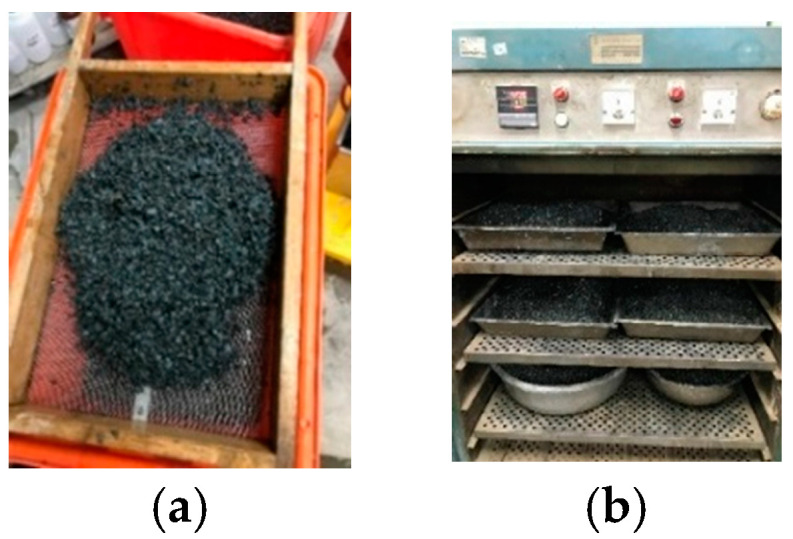
Processing of LWAs after immersion in a nutrient solution: (**a**) draining and (**b**) drying.

**Figure 8:**
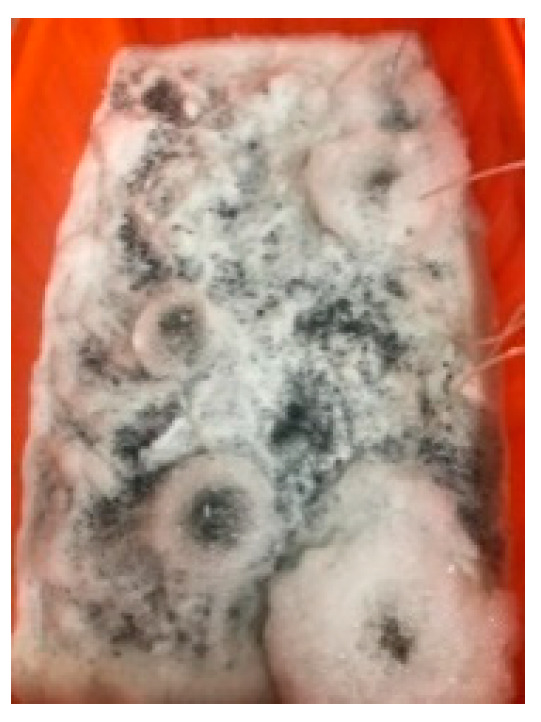
Image of LWAs immersed in a bacterial solution.

Details of the strain culture and sporulation can be found in the author’s previously published articles [44,49]. The LWAs in the experimental group were used as strain carriers. The steps for implanting the strains into the LWAs were as follows:

(a) The LWAs were washed with clean water. Afterwards, the LWAs were dried to a dry state. Then, the LWAs were immersed in a nutrient source solution containing calcium lactate (80 g/L) and yeast extract (1 g/L) for 60 min. The samples were stirred every 10 min, as shown in Figure 6.
(b) The soaked LWA was taken out and drained. Then, the drained LWA was evenly spread on the iron plate and placed in an oven at a constant temperature of 37 °C to dry for 5 days, as shown in Figure 7.
(c) The previous two steps were repeated once.
(d) The nutrient-containing LWAs were immersed in the bacterial spore solution for 60 min, during which the pump continued to run and stir every 10 min, as shown in Figure 8.
(e) After the LWAs were soaked, they were taken out and drained, spread on an iron plate, and then placed in an oven at 37 °C to dry for five days.

### 2.3. The Mix Proportions of LWAC

The 28-day design compressive strength was 35 MPa for both groups of LWAC, and their required compressive strength was 45 MPa for both groups. According to ACI 211.2, considering the workability, strength, and durability of the two groups of LWAC, the dosage of each material was determined through trial mixing, as shown in Table 6. When mixing the LWAC mixture, the saturated-surface dry fine aggregate and cement were first placed into the mixing drum and dry-mixed thoroughly for one minute at a speed of 140 ± 5 rpm. After that, the steel fibers and polypropylene fibers were sprinkled evenly into the mixing drum by hand, and dry-mixed thoroughly for three minutes until they were uniform. Afterwards, the dry LWAs were poured into the mixing drum and stirred at a speed of 285 ± 10 rpm for one minute. Then, the pre-mixed water and superplasticizer were poured into the mixing drum and mixed thoroughly until a homogeneous fresh concrete was formed.

### 2.4. The Casting and Curing of Specimens

**Figure 9:**
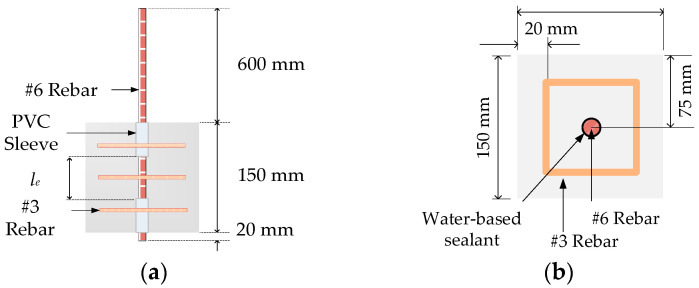
Dimensions and cross-sections of the pull-out specimens: (**a**) side view and (**b**) top view.

The compression test used a cylindrical specimen with a diameter of 100 mm and a height of 200 mm. The pull-out test used a cubic specimen with a side length of 150 mm. A #6 steel bar with a diameter of 19.1 mm was embedded in the central axis of the pull-out specimen, and its embedded length (*l_e_*) was three times the diameter of the steel bar (*d_b_*), as shown in Figure 9. In addition, three transverse stirrups were configured to prevent the splitting failure of the specimen when the longitudinal steel bar was tensile. When casting the pull-out specimens, the non-bonded regions of the steel bar were wrapped with PVC sleeves. To prevent the steel bar of the specimen from being corroded during the curing process, water-based sealant was used to fill the gap between the PVC sleeves and the steel bar. After the mixing of each LWAC mixture was completed, the slump was measured immediately. Then, nine cylindrical specimens and nine pull-out specimens were cast for each group of LWAC mixtures. After twenty-four hours, the specimens were removed from the mold. Then, all specimens were placed in a saturated lime water tank in a curing room. On the 14th day, the specimens were taken out and placed in a 40 °C incubator to cure until 28 days.

**Figure 10:**
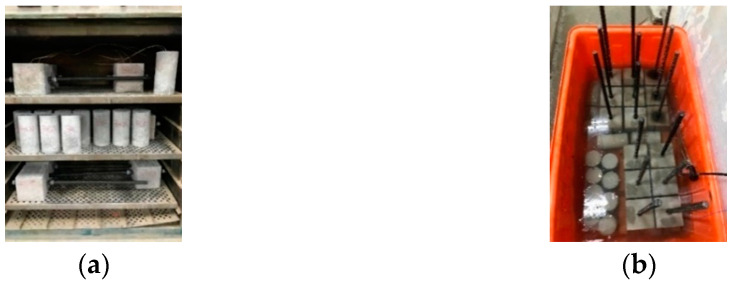
Curing condition of the specimens: (**a**) in an incubator and (**b**) in a curing solution tank.

After casting and curing for 28 days, the two groups of LWAC specimens were subjected to compressive strength and pull-out tests. Then, the two groups of specimens damaged by the tests were subjected to different self-healing methods. The healing method of the control group was to place the specimens in a 40 °C incubator until the test age, as shown in Figure 10a. The experimental group was divided into experimental group I and experimental group II according to the self-healing method. The healing method of experimental group I was the same as that of the control group. The healing method of experimental group II was to immerse the specimen in a mixed solution (1 molar concentration of urea and 0.5 molar concentration of calcium acetate) for two days, as shown in Figure 10b, and then take it out and put it in a 40 °C incubator for two days, with one cycle lasting four days. After reaching the planned age, the specimens were subjected to secondary compressive strength and pull-out tests. The crack healing was also observed, and the composition of the repair compound was identified. The experimental plan of this study is shown in Table 7. The test items included a compressive strength test, a pull-out test, crack repair observation, field emission scanning electron microscope (FESEM) observation, an X-ray energy spectrometer (EDS) analysis, and an X-ray diffraction (XRD) analysis. The test parameters include self-healing method and age.

### 2.5. Test Methods and Data Analysis

**Figure 11:**
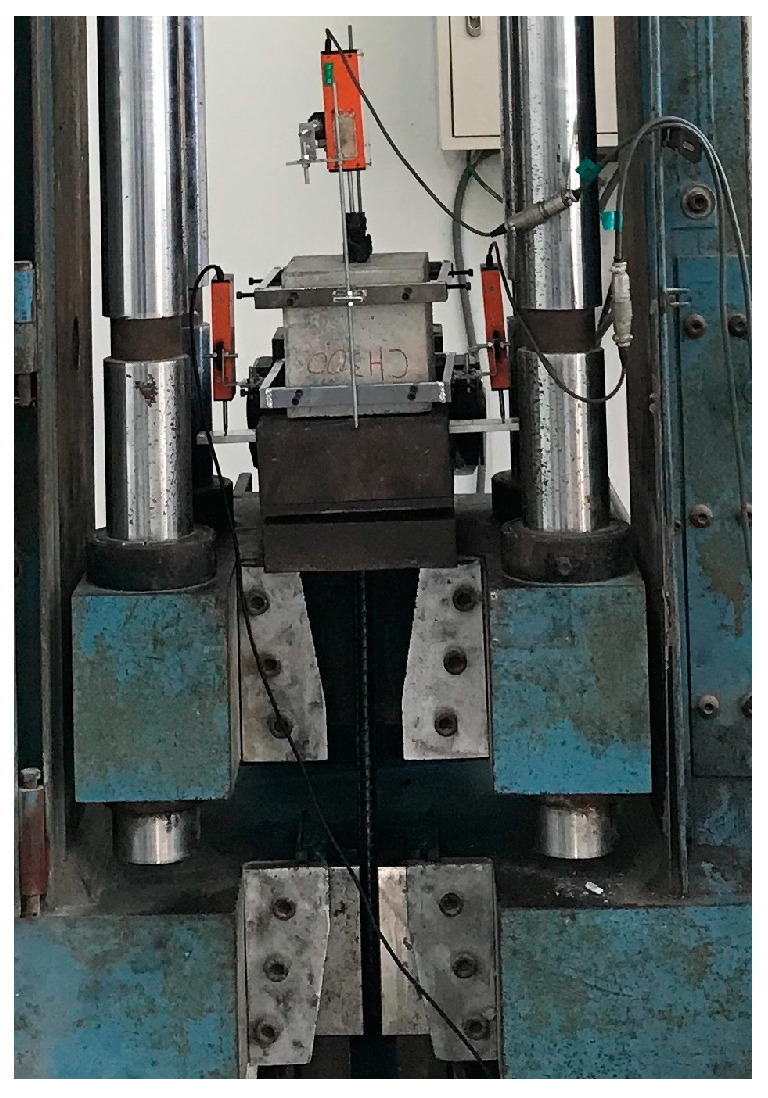
Setup of the pull-out test.

The testing methods for the various properties of concrete in this study were based on the ASTM specifications [52,53,54,55,56]. Three specimens were prepared for each test, and the average value was taken. The compression test and elastic modulus test were conducted according to ASTM C39 [54] and ASTM C469 [55], respectively. The pull-out test was performed in accordance with the specifications of ASTM C234 [56]. The load was applied to the pull-out specimen using a 200 kN MTS servo valve-controlled machine equipped with a special test frame, as shown in Figure 11. In the pull-out specimen, one end of the steel bar is the load end, and the other end is the free end. Through the installation of three linear variable differential transformers (LVDT), the relative bond–slip between the steel bars and concrete at the load end and free end was measured. The transmission lines of the load and LVDT were connected to the data acquisition device, and the test was carried out after zeroing. During the pull-out test, the load was applied at a constant displacement rate of 0.01 mm/s.

Through the pull-out test, the corresponding slip of the steel bars of the specimen under different loads was measured. The bond stress under different loads was obtained from the internal force balance of the specimen shown in Figure 9a [4]:

(5) $$\tau =\frac{P}{\pi d_{b}l_{e}}$$

where τ is the bond stress; *P* is the applied load; $d_{b}$ is the rebar diameter; and $l_{e}$ is the embedded length. The relative slip between the steel bars and concrete corresponding to the bond stress can be divided into loading end slip ($s_{l}$) and free end slip ($s_{f}$). The average value of $s_{l}$ and $s_{f}$ was regarded as the slip corresponding to the bond stress, as shown in the following equation:

(6) $$s=\frac{s_{l}+s_{f}}{2}$$

The tests of the two groups of LWAC were divided into four types, as shown in Table 8. After the compression test was completed, samples were taken from two different parts of the two groups of cylindrical specimens (two centimeters away from the surface and the center). The purpose was to use FESEM and EDS to observe and analyze the microstructure of each group of concrete specimens after self-repair. The samples were cleaned, dried, and gold-plated before FESEM and EDS analysis. In addition, X-ray diffraction analysis was also used to conduct qualitative and semi-quantitative analyses of the element types and contents of each group of concrete samples.

## 3. Experimental Results and Discussions

### 3.1. The Results of the Fresh Properties Test

The slumps of the two groups of LWAC were both 13 cm, and both had good workability. In addition, the unit weight of both groups of LWAC was 1849 kg/m^3^, which was only 80.4% of that of ordinary NWC.

### 3.2. The Results of the Compression Test

#### 3.2.1. The Compressive Strength and Elastic Modulus of the First Compression Test

Table 9 lists the 28-day compressive strength test results of the two groups of LWAC. The values were very close to the required compressive strength (i.e., 45 MPa). As can be seen from Table 9, the 28-day compressive strength of the two groups of LWAC was the same, ranging from 44.59 to 45.88 MPa. Among them, experimental group II had the highest value of 45.88 MPa, followed by experimental group I at 44.81 MPa, and the control group had the lowest value at 44.59 MPa. The structural efficiency (strength/density) of the two groups of LWAC was 24.3 MPa/(t/m^3^), which was within the range of most LWACs, that is, 10–40 MPa/(t/m^3^) [57]. In addition, the 28-day elastic modulus of the two groups of LWAC was roughly similar, ranging from 18.75 to 19.26 GPa. Among them, experimental group II had the highest value of 19.26 GPa, followed by experimental group I at 19.09 GPa, and the control group had the lowest value of 18.75 GPa. The elastic modulus of typical expanded clay LWA was in the range of 10–20 GPa [58,59]. Therefore, the results are consistent with the literature.

#### 3.2.2. The Compressive Strength of the Secondary Compression Test

The cylindrical specimens of the two groups of LWAC after the compression test self-healed in different ways. As can be seen from Table 9, after 28 days of self-healing, the secondary compressive strengths of the control group, experimental group I, and experimental group II were 13.83, 14.37, and 15.61 MPa, respectively. In addition, the relative compressive strength ratio is defined as the ratio of the secondary compressive strength after self-healing to the original 28-day compressive strength. As can be clearly seen from Table 9, the relative compressive strength ratios of the control group, experimental group I, and experimental group II after 28 days of self-healing were 0.31, 0.32, and 0.34, respectively. Compared with the control group, the relative compressive strength ratios of experimental groups I and II increased by 3.2% and 9.7%, respectively. This shows that the self-healing method of experimental group II had a better effect.

### 3.3. The Results of the Pull-Out Test

#### 3.3.1. The Bond Strength of the First Pull-Out Test

The failure modes of the pull-out specimens in both groups of LWAC were shear pull-out failures. This test was characterized by the center steel bar being pulled out of the concrete and a small number of cracks appearing in the surrounding concrete. Based on the data captured during the pull-out test, the maximum bond stress (i.e., bond strength) of the pull-out specimen could be analyzed. The first pull-out test results for each group of specimens are shown in Table 10. As can be seen from Table 10, the 28-day average bond strength of both groups of LWACs was close to 28 MPa. This result exceeds the recommended value of Mo et al. [21] ($3.5\sqrt{f_{c}^{\prime }}$) and the recommended value of the CEB-FIP standard (2$.5\sqrt{f_{c}^{\prime }}$) [24]. This is because LWA has a stronger aggregate interlocking effect and the content of binder material in LWAC is higher, which improves the quality of the cement slurry and, thus, improves the mechanical interlocking performance [21,23]. This result is consistent with the results of Kevinly et al. [60].

#### 3.3.2. The Bond Strength of the Secondary Pull-Out Test

The two groups of LWAC specimens after the pull-out test self-healed in different ways. As can be seen from Table 10, after 28 days of self-healing, the secondary bond strengths of the control group, experimental group I, and experimental group II were 18.84, 20.82, and 22.25 MPa, respectively. In addition, the relative bond strength ratio is defined as the ratio of the secondary bond strength after self-healing to the original 28-day bond strength. As can be clearly seen from Table 10, the relative bond strength ratios of the control group, experimental group I, and experimental group II after 28 days of self-healing were 0.67, 0.73, and 0.79, respectively. Compared with the control group, the relative bond strength ratios of experimental groups I and II increased by 9% and 17.9%, respectively. This shows that after 28 days of self-healing, experimental group II had the highest relative bond strength ratio. Once again, this showed that the self-healing method used in experimental group II had a better effect.

### 3.4. The Local Bond Stress–Slip Relationship of Steel Bars in LWAC

#### 3.4.1. Bond Stress–Slip Relationship in the First Pull-Out Test of Steel Bars in LWAC

**Figure 12:**
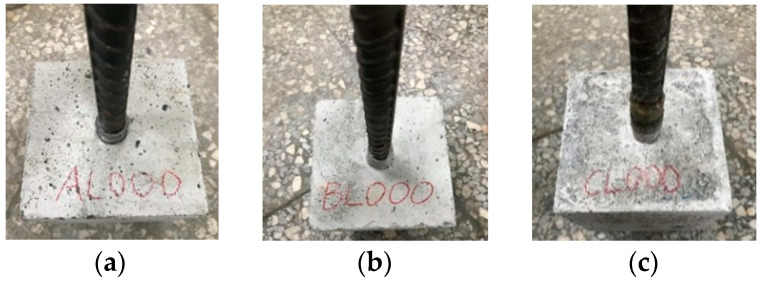
Damage conditions of the LWAC specimen in the first pull-out test: (**a**) control group, (**b**) experimental group I, and (**c**) experimental group II.

Due to the lower strength of LWA, the bond behavior between LWAC and steel bars is different from that between NWC and steel bars [12]. However, there is a lack of a unified understanding of this difference. Therefore, the current research on the bond stress–slip relationship between LWAC and steel bars is mostly determined through experiments [4,19,21]. An analysis of the literature data shows [61,62] that the ratio of concrete protective layer to steel bar diameter (*C*/*D*) in the range of 2.5–3.0 can ensure that the failure mode is pull-out failure. The concrete protective layer of the pull-out specimen in this study was relatively thick (6.5 cm), and its *C*/*D* ratio was 3.4. Furthermore, the specimen had transverse stirrups. As a result, the longitudinal steel bar of the specimen was subject to greater confinement, and the concrete between the transverse bars was sheared due to punching, causing the steel bars to be pulled out directly from the concrete. In other words, all specimens showed a pull-out failure mode, as shown in Figure 12.

**Figure 13:**
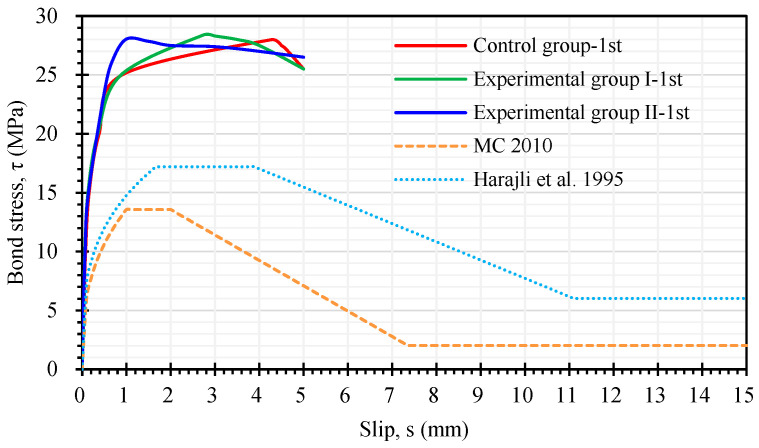
Comparison of the first local bond stress–slip curve with the prediction models [24,32].

In the first pull-out test, the test was stopped when the steel bar slipped 5 mm to avoid further damage. In the subsequent analysis, the first pull-out test of each group of specimens only focused on the slip of the steel bars in the range of 0–5 mm. As can be seen from Figure 13, during the test process, the slip relationship curves of each group of specimens experienced linear ascending, non-linear ascending, and descending stages. In the linear ascending stage, the specimen was not cracked and was in an elastic state; in the non-linear ascending stage, the cracks in the specimen gradually expanded; in the descending stage, the specimen showed obvious cracks. It is worth noting that the ascending branch of the bond stress–slip curve of the two groups of LWAC had a more linear relationship. This is because LWAC’s cementitious matrix provided better chemical adhesion and greater tensile strength. This is consistent with the literature [4]. In addition, the ultimate bond stress of the first pull-out test of the experimental group was slightly higher than that of the control group, but the corresponding slip amount *s*_1_ was smaller, with a value of about 1–3 mm. As can be seen from Figure 13, the ultimate bond stress of the two groups of LWAC was significantly higher than the predicted values of the models of MC 2010 [24] and Harajli et al. [32]. This means that the models of MC 2010 [24] and Harajli et al. [32] are too conservative for estimating the ultimate bond stress of LWAC.

#### 3.4.2. Bond Stress–Slip Relationship in the Secondary Pull-Out Test of Steel Bars in LWAC

**Figure 14:**
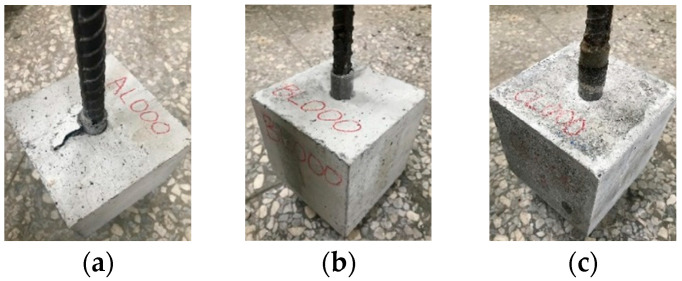
Damage conditions of the LWAC specimens in the secondary pull-out test: (**a**) control group, (**b**) experimental group I, and (**c**) experimental group II.

According to the planned self-curing method, the pull-out specimens that had undergone shear pull-out failure were cured. After the two groups of LWAC specimens had self-cured for 28 days, the secondary pull-out test was conducted. In the secondary pull-out test of the specimen, the test did not stop even if the slip of the steel bar reached 5 mm. Instead, the test continued until the specimen failed completely, as shown in Figure 14.

**Figure 15:**
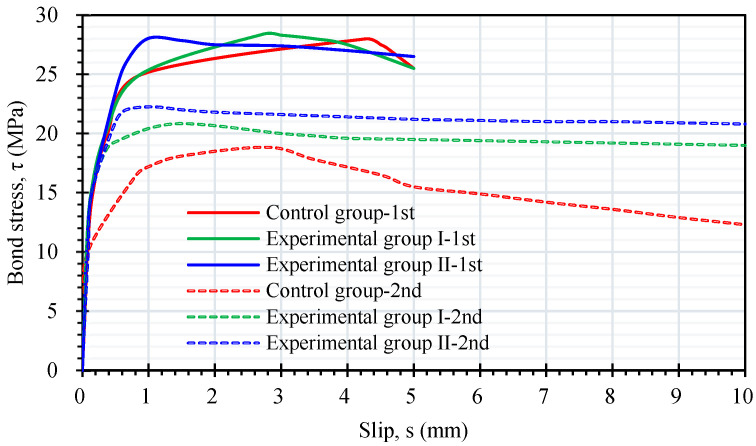
Comparison of the local bond stress–slip relationships between the first and secondary pull-out tests.

Figure 15 shows that the two groups of LWAC specimens still exhibited linear ascending and non-linear ascending stages after self-healing. In addition, there was a plateau section where the bond stress decreased slowly, but the slip continued to increase. Subsequently, a more obvious attenuation occurred. In the residual stage, LWAC generated bond stress through friction. In other words, the bond stress–slip relationship in the secondary pull-out test of the two groups of LWAC specimens was divided into four stages: linear ascending, non-linear ascending, descending, and residual stages, as shown in CEB-FIP 2000 [24]. Comparing the bond stress–slip curves in the first and secondary pull-out tests of the two groups of specimens shows that the ultimate bond stress in the secondary pull-out test was significantly reduced.

**Figure 16:**
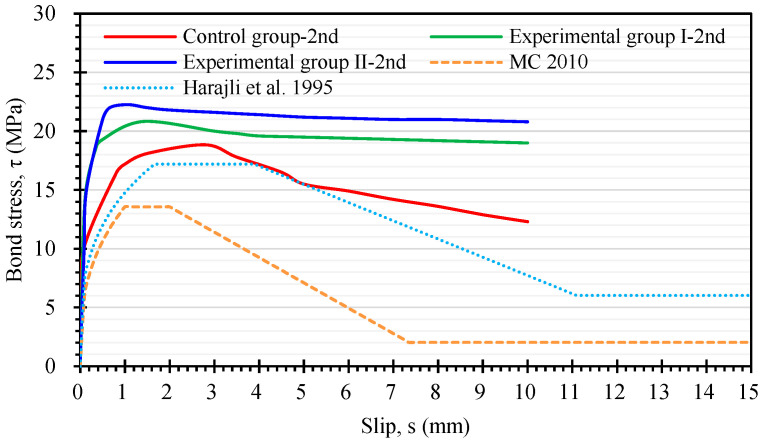
Comparison of the secondary local bond stress–slip curve with the prediction models [24,32].

A comparison of the local bond stress–slip relationship curve obtained from the secondary pull-out test with the prediction model is shown in Figure 16. As can be seen from Figure 16, the ultimate bond stress of the two groups of LWAC was still higher than the predicted value of the models of MC 2010 [24] and Harajli et al. [32]. It is worth noting that the ultimate bond stress of the experimental group specimens in the secondary pull-out test was significantly higher than that of the control group. In particular, the ultimate bond stress of the specimens in experimental group II was 17.9% higher than that of the control group specimens. This is due to the different maintenance environments for each group of specimens.

### 3.5. The Results of the Concrete Crack Healing Observation

**Figure 17:**
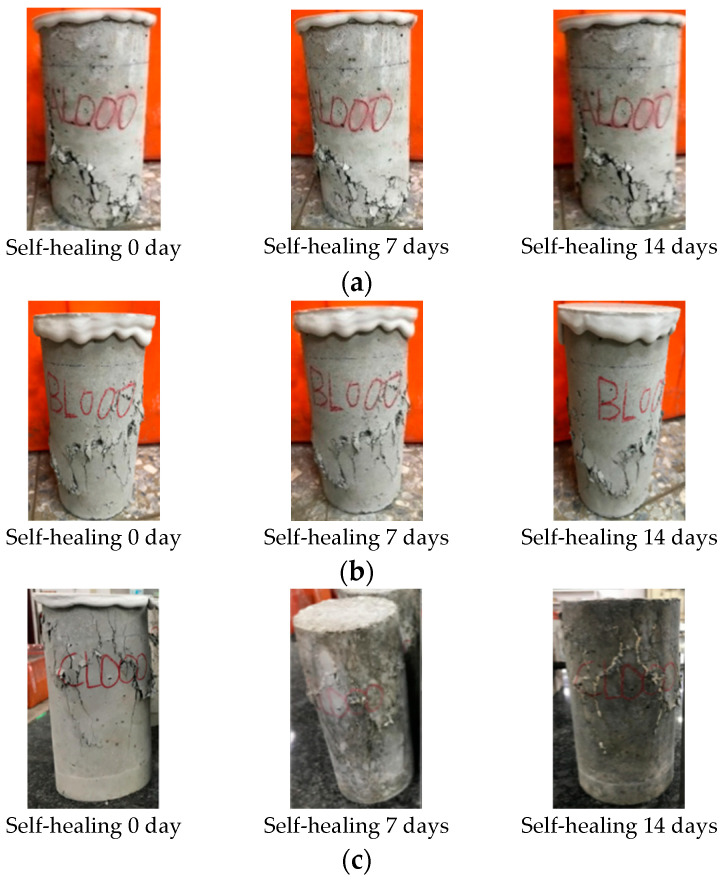
Self-healing of cracks in cylindrical specimens: (**a**) control group, (**b**) experimental group I, and (**c**) experimental group II.

For the cylindrical specimen, the self-healing of the cracks formed in the compression test was observed. The size and location of these cracks were difficult to control. Therefore, only cracks on the surface were observed, as shown in Figure 17. For the specimens in the control group and experimental group I, as the age increased, the self-healing of the cracks was not obvious, as shown in Figure 17a,b. In contrast, starting on the seventh day, calcium carbonate crystals began to crystallize in the cracks on the surface of the experimental group II specimen, as shown in Figure 17c. Moreover, as the curing period increased, the crystals tended to become denser and more extensive. This result showed that the self-healing effect of cracks in each group of specimens was closely related to the curing environment.

### 3.6. The Results of FESEM Images, EDS Analysis, and XRD Analysis

#### 3.6.1. The Results of FESEM Images

**Figure 18:**
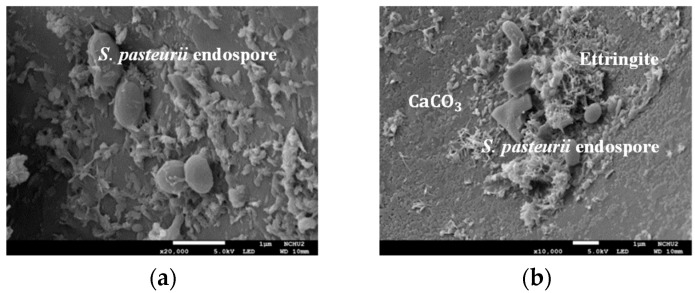
FESEM images of the experimental group sample with a self-healing age of 0 days (**a**) taken from the surface block and (**b**) taken from the center block.

**Figure 19:**
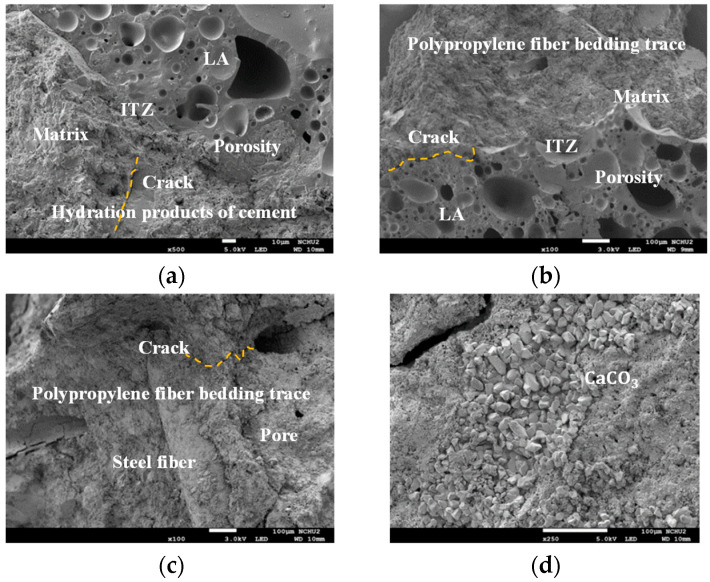
FESEM images of the surface blocks of each group of samples with a self-healing age of 28 days: (**a**) control group, (**b**) experimental group I, (**c**) experimental group II (100× magnification), and (**d**) experimental group II (250× magnification).

After the first compression test, samples were taken at 2 cm from the surface of the cylindrical specimen and at the center. The endospore of *S. pasteurii* bacteria was observed near the surface and in the center of the specimen, as shown in Figure 18. In addition, calcium carbonate blocks appeared around the strains. Moreover, after 28 days of the self-healing of the cylindrical specimen, samples were also taken at 2 cm from the surface of the cylindrical specimen and at the center. Figure 19 shows the FESEM images of the two groups of LWAC samples after 28 days of self-healing. As can be seen from Figure 19, the ITZ of the control group and experimental group I had the same density. Many calcium carbonate crystal particles with parallel polygonal cubic structures were found on the surface of the block in experimental group II. In other words, there were obvious white calcite clusters in the experimental group II samples. This result confirmed that the samples of experimental group II had microbially induced calcium carbonate precipitation (MICP) [63]. During the MICP process, the metabolic activity of microorganisms increased the local saturation state of bacterial cells. This promoted the precipitation of CaCO_3_ [64,65,66]. Therefore, experimental group II could achieve better repair and healing effects, thus improving its performance.

#### 3.6.2. The Results of EDS Analysis

**Figure 20:**
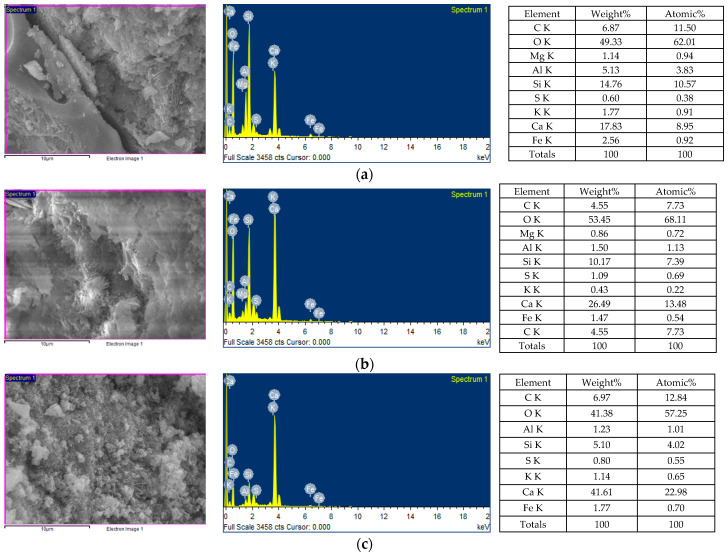
EDS analysis results of each group of samples with a self-healing period of 28 days: (**a**) control group, (**b**) experimental group I, and (**c**) experimental group II.

The EDS test was performed at the interface between the LWA and the matrix of the FESEM-observed sample that had been left to self-heal for 28 days. The EDS spectra of each group of samples are shown in Figure 20. From the EDS analysis, the elements contained in each group of samples were C, O, Mg, Al, Si, K, Ca, Fe, S, Na, and other elements. It is worth noting that in the precipitation-filled crack region of the experimental group II sample, Ca, C, and O bonded to form CaCO_3_. This result confirmed that microbial mineralization occurred in the experimental group II samples [63]. Therefore, experimental group II achieved better performance compared to the control group.

#### 3.6.3. The Results of XRD Analysis

**Figure 21:**
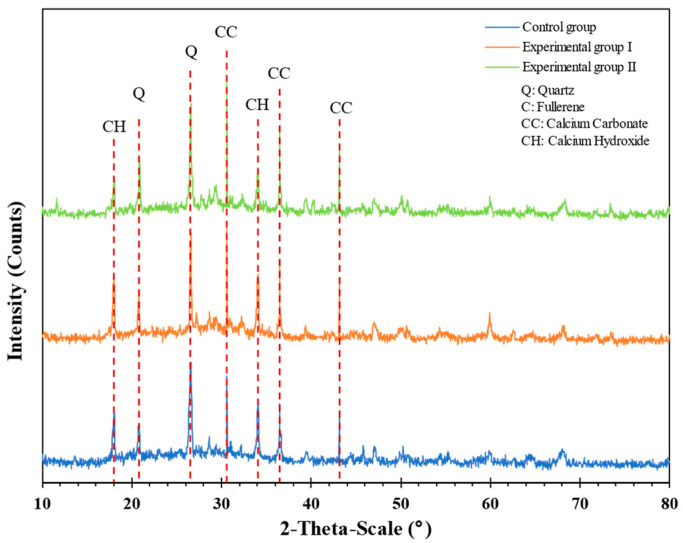
XRD analysis of each group of samples with a self-healing period of 28 days.

X-ray diffraction (XRD) analysis was performed on the LWAC samples to determine their mineral composition. In comparison with the material crystal standard card of the inorganic crystal structure database (ICSD) and the crystallographic information file (CIF) of the material, the XRD analysis results of each group of samples were obtained, as shown in Figure 21. As can be seen from Figure 21, the main reflection angles (2-theta) of the calcium carbonate crystal (reference code: 01-086-2343) were 30.6°, 36.5°, and 43.1°. In addition, the X-ray reflection energy intensity of the experimental group at all angles was higher than that of the control group. Quartz crystal (reference code: 01-089-3755) had main reflection angles (2-theta) of 20.8° and 26.5°. The main reflection angles of calcium hydroxide crystals (reference code: 01-089-1263) were (2-theta) 18.0° and 34.1°. The X-ray reflected energy intensity at all angles in the experimental group was lower than that in the control group. This indicated that the addition of the *S. pasteurii* strain could effectively reduce the formation of calcium hydroxide (CH) crystals. In addition, it could be converted into cubic crystals and agglomerated materials composed of other structures. Due to the poor composition of the CH crystal structure, its contribution to strength is low. This can verify that experimental group II in the secondary compression test and the secondary pull-out test of this study did have higher strength.

## 4. Conclusions

The self-healing of concrete cracks can improve the concrete’s durability and sustainability, thereby extending the service life of concrete structures. This study applied biomineralization to improve damaged fiber-reinforced LWAC. After the damaged specimen healed itself for 28 days, the secondary compression test and the secondary pull-out test were conducted. The relative compressive strength ratios of the control group, experimental group I, and experimental group II were 0.31, 0.32, and 0.34, respectively. Compared with the control group, the relative compressive strength ratio of experimental groups I and II increased by 3.2% and 9.7%, respectively. However, the relative bond strength ratios of the control group, experimental group I, and experimental group II were 0.67, 0.73, and 0.79, respectively. In particular, the ultimate bond stress ratio of the experimental group II was significantly higher. Compared with the control group, the relative bond strength ratios of the experimental groups I and II increased by 9% and 17.9%, respectively. Moreover, the precipitate formed at the cracks in the sample was confirmed to be calcium carbonate with the EDS and XRD analysis results, which improved the compressive strength and bond strength after self-healing. This indicates that the biomineralization self-healing method used in experimental group II is more effective.

## Figures and Tables

**Table 1:** Parameter values for the prediction models for the bond stress–slip relationship.

Parameter	Model Code 2010 (2010)		Harajli et al. (1995) [32]
	Confined NWC	Confined LWAC	Concrete
$s_{1}$	1.0 mm	1.0 mm	0.15 Distance bet. ribs
$s_{2}$	3.0 mm	2.0 mm	0.35 Distance bet. ribs
$s_{3}$	Clear rib spacing	Clear rib spacing	Distance bet. ribs
α	0.4	0.35	0.3
$\tau_{u}$	$2.5\sqrt{f_{c}^{\prime }}$	$0.6(f_{c}^{\prime })$0.82	$2.57\sqrt{f_{c}^{\prime }}$
$\tau_{f}$	$0.4\tau_{u}$	$0.15\tau_{u}$	$0.9\sqrt{f_{c}^{\prime }}$

**Table 2:** Chemical compositions of cement.

Chemical Composition of Cement	Weight%
Silicon dioxide, SiO_2_	20.22
Aluminum oxide, Al_2_O_3_	5.85
Iron oxide, Fe_2_O_3_	3.35
Calcium oxide, CaO	64.67
Magnesium oxide, MgO	2.03
Sulfur trioxide, SO_3_	2.36
Free calcium oxide, f-CaO	0.77
Loss on ignition, LOI	0.75
Tricalcium silicate, C_3_S	59.50
Dicalcium silicate, C_2_S	13.83
Tricalcium aluminate, C_3_A	9.98

**Table 3:** Basic properties of LWAs.

Items	State of LWAs	
	With Bacterial Spores	Without Bacterial Spores
Dry unit weight (kg/m^3^)	622.1 (18.5)	618.8 (21.4)
Porosity (%)	622.7 (21.3)	619.6 (22.1)
Bulk specific gravity	47.53 (2.4)	45.22 (1.8)
Apparent gravity	1.188 (0.042)	1.172 (0.045)
1-h water absorption rate (%)	1.234 (0.047)	1.225 (0.054)
24-h water absorption rate (%)	1.246 (0.058)	1.233 (0.063)
Crushing strength (MPa)	8.6 (0.408)	11.4 (0.435)

Note: The data in brackets are standard deviations.

**Table 4:** Basic properties of fibers.

Fiber Type	Length (mm)	Diameter (mm)	Density (g/cm^3^)	Elastic Modulus (GPa)	Tensile Strength (MPa)	Melting Point (°C)
SF	13	0.2	7.8	200	2000	-
PP	12	0.05	0.9	-	300	165

Notes: SF: steel fiber; PP: polypropylene fiber.

**Table 5:** Basic properties of rebar.

Nominal Dia. (mm)	Rib Distance (mm)	Rib Width (mm)	Rib Height (mm)	Yield Strength (MPa)	Tensile Strength (MPa)
19.1	11.1	4.0	1.0	457	658

**Table 6:** Mix proportions of the concretes.

Group	W/B	W (kg/m^3^)	C (kg/m^3^)	LWA (kg/m^3^)	FA (kg/m^3^)	SF (kg/m^3^)	PP (kg/m^3^)	SP (kg/m^3^)
Control group	0.45	220	489	345	734	58.5	1.17	0.978
Experimental group								

Notes: W/B: water–binder ratio; W: water; C: cement; LWA: lightweight aggregate; FA: fine aggregate; SF: steel fiber; PP: polypropylene fiber; SP: superplasticizer.

**Table 7:** Test items and test parameters.

Test Items	Test Parameters	
	Curing/Healing Method	Self-Healing Age (Day)
Compressive strength test	Incubator, water tank	0
Pull-out test	Incubator, water tank	0
Secondary compressive strength test	Incubator, cyclical treatment	28
Secondary pull-out test	Incubator, cyclical treatment	28
Observation of crack repair	Incubator, cyclical treatment	0, 7, 14
FESEM, EDS, and XRD analysis	Incubator, cyclical treatment	0, 28

**Table 8:** Test items and test sequences.

Test Item	Test Sequence
Compression test after 28 days of curing	Curing→loading
Secondary compression test after self-healing of compressive failure specimen	Curing→loading→self-healing→reloading
Pull-out test after 28 days of curing	Curing→loading
Secondary pull-out test after self-healing of pull-out failure specimen	Curing→loading→self-healing→reloading

**Table 9:** Test results of the compressive strength and elastic modulus of the LWACs.

Group	Elastic Modulus (GPa)	First Compressive Strength (MPa)	Secondary Compressive Strength after Self-Healing (MPa)	Relative Compressive Strength Ratio after Self-Healing
Control group	18.75 (0.56)	44.59 (1.42)	13.83 (0.39)	0.31
Experimental group I	19.09 (0.55)	44.81 (1.34)	14.37 (0.45)	0.32
Experimental group II	19.26 (0.60)	45.88 (1.33)	15.61 (0.47)	0.34

Note: The data in brackets are standard deviations.

**Table 10:** Results of the pull-out test.

Group	First Bond Strength (MPa)	Secondary Bond Strength (MPa)	Relative Bond Strength Ratio
Control group	27.99 (1.56)	18.84 (0.86)	0.67
Experimental group I	28.38 (1.34)	20.82 (0.37)	0.73
Experimental group II	28.02 (0.40)	22.25 (1.01)	0.79

Note: The data in brackets are standard deviations.

## Data Availability

The data presented in this study are available upon request from the corresponding author.
